# Quantifying Insulin Sensitivity and Entero-Insular Responsiveness to Hyper- and Hypoglycemia in Ferrets

**DOI:** 10.1371/journal.pone.0090519

**Published:** 2014-03-03

**Authors:** Hongshu Sui, Yaling Yi, Jianrong Yao, Bo Liang, Xingshen Sun, Shanming Hu, Aliye Uc, Deborah J. Nelson, Katie Larson Ode, Louis H. Philipson, John F. Engelhardt, Andrew W. Norris

**Affiliations:** 1 Department of Anatomy and Cell Biology, University of Iowa Carver College of Medicine, Iowa City, Iowa, United States of America; 2 Department of Pediatrics, University of Iowa Carver College of Medicine, Iowa City, Iowa, United States of America; 3 Department of Histology and Embryology, Taishan Medical University, Taian Shandong, China; 4 Department of Neurobiology, Pharmacology & Physiology, University of Chicago, Chicago, Illinois, United States of America; 5 Department of Medicine, University of Chicago, Chicago, Illinois, United States of America; 6 Fraternal Order of Eagles Diabetes Research Center, University of Iowa, Iowa City, Iowa, United States of America; Virginia Tech, United States of America

## Abstract

Ferrets are an important emerging model of cystic fibrosis related diabetes. However, there is little documented experience in the use of advanced techniques to quantify aspects of diabetes pathophysiology in the ferret. Glycemic clamps are the gold standard technique to assess both insulin sensitivity and insulin secretion in humans and animal models of diabetes. We therefore sought to develop techniques for glycemic clamps in ferrets. To assess insulin sensitivity, we performed euglycemic hyperinsulinemic clamps in 5–6 week old ferrets in the anesthetized and conscious states. To assess insulin secretion, we performed hyperglycemic clamps in conscious ferrets. To evaluate responsiveness of ferret islet and entero-insular hormones to low glucose, a portion of the hyperglycemic clamps were followed by a hypoglycemic clamp. The euglycemic hyperinsulinemic clamps demonstrated insulin responsiveness in ferrets similar to that previously observed in humans and rats. The anesthetic isoflurane induced marked insulin resistance, whereas lipid emulsion induced mild insulin resistance. In conscious ferrets, glucose appearance was largely suppressed at 4 mU/kg/min insulin infusion, whereas glucose disposal was progressively increased at 4 and 20 mU/kg/min insulin. Hyperglycemic clamp induced first phase insulin secretion. Hypoglycemia induced a rapid diminishment of insulin, as well as a rise in glucagon and pancreatic polypeptide levels. The incretins GLP-1 and GIP were affected minimally by hyperglycemic and hypoglycemic clamp. These techniques will prove useful in better defining the pathophysiology in ferrets with cystic fibrosis related diabetes.

## Introduction

Ferrets (*Mustela putorius furo*) are a domestic mammal. Ferrets are excellent models of a wide variety of human diseases including influenza, lung disease, cardiovascular disease, and peptic ulcer disease [1]. Within the past year, the sequencing of the ferret genome was completed, rendering the molecular tool-box for ferret research readily accessible. Furthermore, genetic engineering of ferret models of human disease is now possible. We recently described ferrets with targeted knockout of the cystic fibrosis transmembrane conductance regulator (CFTR) gene [2]. Like humans with cystic fibrosis (CF), the CFTR(−/−) ferrets develop severe lung and gastrointestinal disease [3] and thus serve as a highly relevant model for unraveling CF pathophysiology.

Nearly 50% of humans with CF develop diabetes by middle age [4]. The etiology of CF-related diabetes (CFRD) is poorly understood, but involves impaired insulin secretion in response to glucose [5]–[7] and reduced insulin sensitivity especially in the liver [8]–[11]. In humans with CF, first phase insulin secretion is diminished regardless of glucose tolerance [6]. Insulin secretion is not absent, however, and late phase insulin secretion is often exaggerated [6]. The mechanisms by which CFTR defects dysregulate insulin secretion are not known. Remarkably, spontaneous episodes of hypoglycemia can complicate CF [12], [13], though the underlying mechanisms are unknown. Because CF induces significant intestinal pathology, it has also been suggested that alterations in gut hormone levels may contribute to insulin secretory dysregulation. Indeed, alterations in gut hormone levels have been described in humans with CF [14]–[17].

Although ferrets occasionally develop spontaneous diabetes [18]–[20], ferrets have not been used extensively as models to study diabetes. The etiology of spontaneous ferret diabetes is uncertain. Ferrets over 3 years of age are also extremely prone to developing insulinomas [19], [21]. We recently reported that CFTR-knockout ferrets, like humans with CF, develop spontaneous diabetes [22]. The onset of spontaneous diabetes in CF ferrets coincides with the age at which exocrine pancreatic disease becomes severe. However, the exact physiologic nature of impaired insulin secretion in the CF ferrets, and the possible contribution of insulin resistance, remain poorly defined. At birth, before structural exocrine pancreas disease, CF-ferrets have defects in insulin secretion despite normal total beta-cell mass and normal glucose levels. Like humans, during glucose tolerance testing CF ferrets have diminished first phase and augmented late phase insulin secretion [22]. Also suggestive of dysregulated insulin secretion was the unexpected finding that random-fed glucose levels were slightly diminished in newborn CF ferrets and associated with marked insulin elevation [22]. This suggests that there may be an inherent dysregulation of insulin secretion in CF ferrets caused by improper suppression of insulin secretion during hypoglycemia and that this phenomenon may contribute to hypoglycemia in humans with CF [12].

These findings demonstrate the importance of the CF ferret in understanding the pathophysiological mechanisms involved in CFRD. To this end, we sought to develop techniques to better define insulin sensitivity and insulin secretion in the ferret. Glycemic clamps are the gold standard to quantify insulin metabolic action and insulin secretion. Euglycemic hyperinsulinemic clamps serve to determine insulin sensitivity [23], whereas hyperglycemic clamps serve to define insulin secretion [24]. Herein, we report development of techniques to perform euglycemic hyperinsulinemic and hyperglycemic clamps in ferrets. Because data in the CF ferret model suggests the potential for impaired shutoff of insulin secretion during hypoglycemia [22], we also developed a clamp protocol capable of assessing the kinetics of insulin reductions following the transition from a hyperglycemic to a hypoglycemia state. We also determined the responsiveness of other islet and gut hormones to hyperglycemia and hypoglycemia. These procedures will ultimately serve not only to better define the physiology of CFRD, but also could be used to better define spontaneous diabetes that occurs in wild-type ferrets.

## Materials and Methods

### Ethics Statement

All ferret procedures herein were performed according to protocols approved by the Institutional Animal Care and Use Committee of the University of Iowa.

### Animals

Wild type ferrets and ferret chow were purchased from Marshall Farms (North Rose, NY). Ferrets were housed in separate cages under controlled temperature (20–22°C) and long day light cycle (16 h light/8 h dark) with free access to water and food. Glycemic clamps were performed on 5–6 week old ferrets.

### Assays

Plasma insulin levels were measured by ELISA kit (IS130D, Calbiotech, Spring Valley, CA), by a customized Milliplex surface fluorescent-coded magnetic bead assay (HMHMAG-34K, Millipore, Billerica, MA), and/or by porcine ELISA kit (Cat #10-1200-01, Mercodia, Uppsala, Sweden). Insulin measurements using the Milliplex and porcine ELISA assays were standardized to responses of the Calbiotech kit by 12 co-measures across a range (1–50 µM/mL) of ferret insulin concentrations. Plasma cortisol was measured using an ELISA kit (CO103S, Calbiotech). Plasma glucagon, PP, GLP-1, and GIP were measured using the same Milliplex assay as used for insulin. Real-time glucose levels were measured on whole blood withdrawn from the arterial catheter using a glucose meter (One Touch, LifeScan Inc.,Milpitas, CA).

### Catheter placement

Vascular catheters were placed in ferrets during anesthesia induced and maintained with inhalational isoflurane mixed with oxygen. Platinum-cured silicone catheters (0.012″ ID, Cat #60985-700, VWR), containing a 500 IU/mL Na⋅heparin 0.63 g/mL glycerol lock solution, were introduced into the right jugular vein and left carotid artery. The free catheter ends were directed subcutaneously to a small exit in mid-back. Ferrets received post-operative care and the catheter was maintained by daily flushing with 200 U/mL heparin in saline.

### Hyperinsulinemic euglycemic clamps

The ferrets were allowed to recover from catheter placement surgery for 4–6 days before undergoing glycemic clamps. Study ferrets gained on average 8.7±3.8% of their body weight in this interval and none lost weight. Clamps were initiated after 5–6 hour fast. The hyperinsulinemic euglycemic clamps were performed in either the anesthetized or the conscious minimally-restrained state. For anesthetized clamps, isoflurane was the anesthetic agent employed. For conscious minimally-restrained clamps, ferrets were maintained in an open plastic cage (12×16×9 in) containing animal bedding. Restraint consisted of a shoulder harness (VAH95AB, Instech, Plymouth Meeting, PA) interfaced to rubber tubing (0.2″ ID). This setup allowed the ferrets free movement within the cage. Silicone tubing (0.015″ ID) was used to connect infusion pumps to the infusion catheter, coursing between the two via the rubber tubing. Infusion solutions were mixed by Y-connector (Instech, SCY22) placed near the ferret to minimize the post-mixing catheter dead volume. Infusions were delivered via the jugular catheter and the carotid catheter was used for blood sampling. After a 30 minute basal sampling period, a continuous infusion of 4 mU/kg/min human regular insulin (Humulin R, Eli Lilly, Indianapolis) at 10 µL/min was provided for 120 minutes. Blood was collected at 10 min intervals for the immediate measurement of blood glucose, and a 20% dextrose solution infused at variable rates using the negative feedback principle to maintain euglycemia at its basal level. Blood samples (100 µl) were taken just prior to and at the conclusion of the insulin infusion (time 0 and 120 min respectively) for plasma hormone measurements. Prior to euglycemic hyperinsulinemic clamp, a portion of the ferrets were treated with triglyceride emulsion and heparin, a treatment that induces insulin resistance in variety of species [25]–[27]. The evening before the clamp, 0.025 mL/g of a 20% triglyceride emulsion (Intralipid, NDC 0338-0519-02) and 0.5 U/g heparin (NDC 25021-400-10) were administered subcutaneously, and repeated 4 hours prior to the clamp.

### Traced Euglycemic Hyperinsulinemic Clamps

A separate set of euglycemic hyperinsulinemic clamps employed a glucose tracer in order to determine glucose production and glucose disposal rates. These clamps were performed in the awake, minimally restrained state using insulin infusion rates of 4 (“I4”) or 20 (“I20”) mU/kg/min. The tracer, 6,6-D_2_-glucose (98% enrichment, Sigma Aldrich, St Louis, MO), was infused at 0.3 or 0.4 mg/kg/min in the I4 and I20 clamps respectively, starting 90 minutes prior to the initiation of insulin infusion. Infusion was initiated with a single bolus of 8 or 10 mg/kg for the I4 and I20 clamps respectively. Plasma samples for determination of mean percent tracer enrichment were collected at −90, −20, −10, 0, 100, 110, and 120 minutes, where time zero is the initiation of insulin infusion. Glucose isotope enrichment was determined by Metabolic Solutions, Inc, (Nashua, NH). Glucose appearance (R_a_) and glucose disposal (R_d_) rates were determined as described [28].

### Hyperglycemic clamp

Hyperglycemic clamps were performed exclusively in conscious minimally-restrained ferrets, using the same procedures as above. At time 0, an infusion of 50 mg/kg/min dextrose was initiated. The rate of dextrose administration was varied thereafter based on real-time blood glucose measurements with a glycemic goal of 240 mg/dL. This glycemic goal was based on extrapolation from human studies [24] as a glucose level likely to induce significant insulin secretion while avoiding marked hyperglycemia. Blood samples (100 µl) were obtained at 0, 2.5, 5, 10, 20, 60, 90, 120, 150, and 180 min for quantification of plasma hormone levels.

### Hypoglycemic clamp

Procedures were established to assess the hormonal responses to the sudden transition from hyperglycemia to hypoglycemia. At 120 minutes during the hyperglycemic clamp, a portion of the ferrets were continued on hyperglycemic infusion, whereas another portion of the ferrets underwent a hypoglycemic phase. In this group of ferrets, the glucose infusion was discontinued at 120 minutes and a continuous infusion of 20 mU/kg/min insulin lispro (Eli Lilly) initiated with an additional 50 mU/kg infused during the initial 5 minutes. Insulin lispro does not cross-react with several assays that detect ferret insulin, as detailed in *Results*. As hypoglycemia ensued, the variable dextrose infusion was resumed with the goal of clamping the blood glucose at 45 mg/dL. The glycemic goal was based on extrapolation from human studies [29] as a glucose level likely to influence islet secretion but avoid hypoglycemia severe enough to risk ferret morbidity. Blood samples (100 µl) were obtained at 120, 125, 130, 150 and 180 min for determination of plasma hormonal concentrations including ferret insulin.

### Statistical Analysis

The statistical significance of differences between two groups was assessed by Student's t-test. Differences between more than two groups were assessed by ANOVA followed by Tukey's HSD post-hoc analysis to assess statistical significance of pair-wise differences. Correlation between groups was assessed by Pearson's product-moment, applying Bonferroni correction for the number of correlations tested. The number of animals per group, N, whose samples were analyzed is shown in figures and tables. In some cases, samples were not available for specific animals for certain assays; these instances are apparent from the listed N.

## Results

### Assessment of Insulin Sensitivity

**Figure 1:**
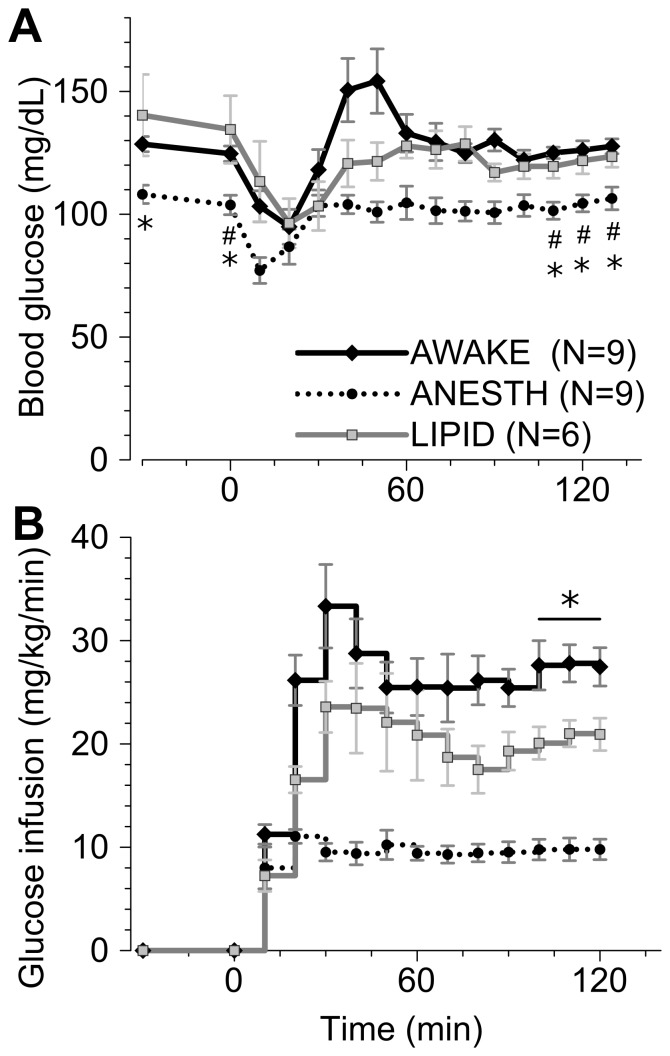
Euglycemic hyperinsulinemic clamp in ferrets. Insulin was infused at 4/kg/min starting at time 0. A portion of the ferrets were anesthetized with isoflurane (ANESTH, dotted line) during the clamp. The remaining ferrets were conscious and minimally restrained, having been treated with triglyceride emulsion (LIPID, grey line) or not (AWAKE, black line). **(A)** Blood glucoses, * p<0.05 versus LIPID, # p<0.05 versus AWAKE. **(B)** glucose infusion rates, * p<0.05 for each group being different. Statistics were only evaluated for the two basal and three final time points.

Euglycemic hyperinsulinemic clamps were performed in ferrets to define their insulin sensitivity. Three conditions were assessed: ferrets anesthetized with isoflurane (ANESTH), conscious and minimally-restrained (AWAKE), and conscious and minimally-restrained treated with triglyceride emulsion and heparin (LIPID). Though the ferrets in the ANESTH group coincidentally weighed less than those in the LIPID group, all three groups had similar basal insulin levels (Table 1). Likewise, basal cortisol levels were similar between groups (Table 1). Euglycemia was maintained throughout the clamp in all three groups, though the ANESTH ferrets had modestly lower blood glucose in the basal and clamp periods (Figure 1A). Serum insulin levels rose significantly during the hyperinsulinemic phase in each group (Table 1), though much higher insulin levels were achieved in the ANESTH compared to other groups. AWAKE ferrets required glucose infusion rates approaching 30 mg/kg/min at steady state during the clamp (Figure 1B). As expected, LIPID ferrets required lower infusion rates to maintain euglycemia (Figure 1B), indicating relative insulin resistance. ANESTH ferrets required the lowest glucose infusion rates, indicating severely diminished insulin responsiveness (Figure 1B).

**Table 1:** Measured parameters during euglycemic hyperinsulinemic clamp, by group.

			Insulin (µU/mL)		Cortisol (µg/dL)	
Group	N	Weight (g)	Fasting	Clamp	Fasting	Clamp
ANESTH	9	162±5 *	4±1^a^	141±27 a ^,^ # ^,^ &	1.7±0.3 b [1.1–3.0]	1.6±0.6 c [0.8–4.8]
AWAKE	9	184±14	7±2	45±9 #	3.4±1.0 [0.1–7.2]	7.1±2.6 [0.8–22]
LIPID	6	223±18	10±8	24±8 #	11±6.8 [1.2–45]	3.5±0.9 [0.9–6.3]

Values shown are the mean ± standard error of measurement and [range] for cortisol. Insulin was measured by Calbiotech ELISA kit.

* p<0.05, versus LIPID;

# p<0.005 versus fasting, paired t-test;

& p<0.005 versus other groups. The N column represents the number of animals studied. For some measures, samples were not available from all animals, in which case the number of samples measured is footnoted.

a N = 8.

b N = 6.

c N = 7.

Mean cortisol levels were statistically unchanged during the clamp and did not differ between groups (Table 1), though there was a trend toward lower levels in the ANESTH group. Two baseline LIPID and two clamp AWAKE cortisol levels exceeded published normative ranges of 0.9–8.5 µg/dL [30]. In general there was not a correlation between cortisol and glucose levels (Supplemental Figure S1A), with the exception that higher end-of-clamp cortisol levels were associated with higher minimum blood glucose levels. Baseline and clamp cortisol levels were not correlated with glucose infusion rates across all ferrets or within individual groups (Supplemental Figure S1B, p = n.s., n = 5–20 pairs).

**Figure 2:**
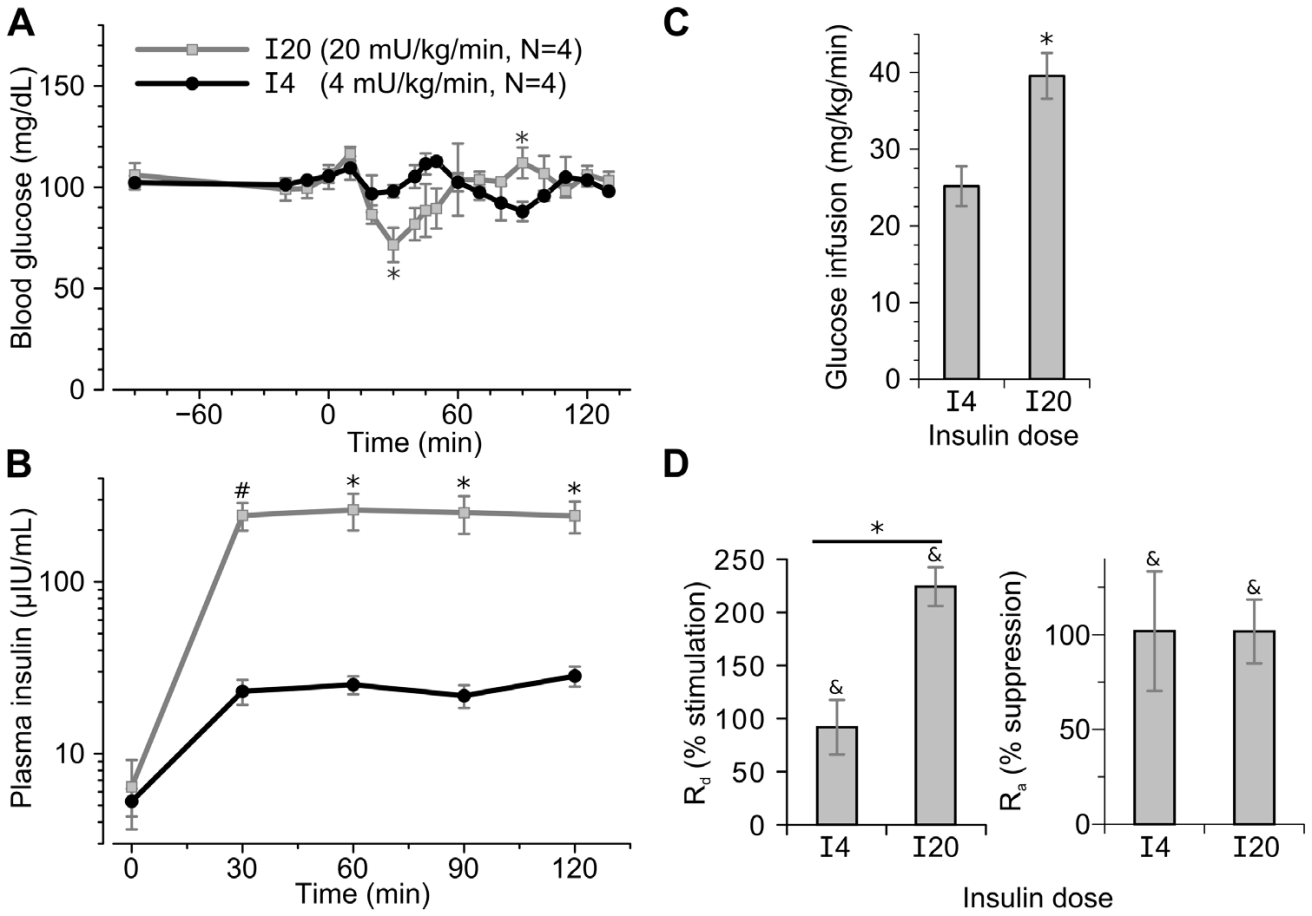
Traced euglycemic hyperinsulinemic clamp at differing insulin doses. Insulin was infused at 4 (“I4”, black lines) or 20 (“I20”, grey lines) mU/kg/min starting at 0 minutes in conscious, minimally restrained ferrets. 6,6-D_2_-glucose was infused throughout the clamp starting at −90 minutes. **(A)** Blood glucose levels during the clamps. **(B)** Plasma insulin levels during the clamps measured by Calbiotech ELISA kit. **(C)** Glucose infusion rates during the final 20 minutes of the two clamps. **(D)** Left panel, stimulation of glucose disposal rates (R_d_) during the clamp as compared to basal rates; right panel, suppression of glucose appearance (R_a_) rates during the clamp as compared to basal rates. Statistics: * p<0.05 I20 versus I4 infusions, # p<0.005 I20 versus I4 infusions; & p<0.05 clamped (100–120 min) rate versus basal (−20–0 min).

To assess insulin dose response, euglycemic hyperinsulinemic clamps were performed at 4 (“I4”) and 20 (“I20”) mU/kg/min insulin infusion rates in conscious ferrets (Figure 2A–D). As expected, I20 clamps induced greater plasma insulin levels compared to I4 clamps (Figure 2B) and required greater glucose infusion rates to maintain euglycemia (Figure 2C). Glucose disposal was approximately doubled by the I4 and more than tripled by I20 insulin infusion (Figure 2D). By contrast, glucose disposal was fully suppressed at both insulin doses.

### Glycemic modulation of insulin levels in ferrets

**Figure 3:**
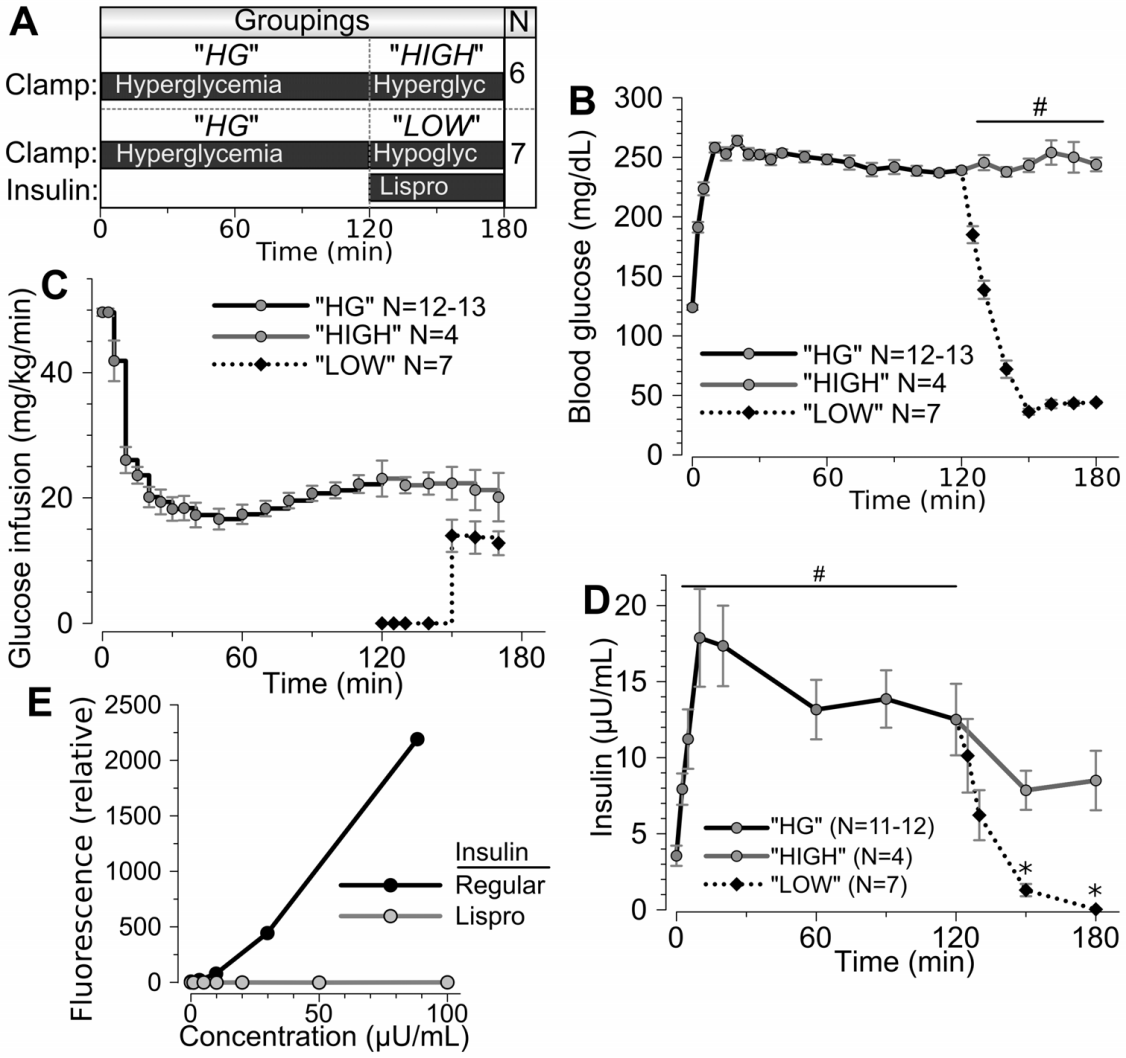
Hyperglycemic clamps in ferrets, also testing hypoglycemic responsiveness. **(A)** All ferrets were subject to 120 minutes of hyperglycemic clamp (HG, N = 13). At 120 min, a portion (LOW, N = 7) of the ferrets underwent insulin lispro infusion and hypoglycemic clamp, while the remaining ferrets (HIGH, N = 6) underwent continued hyperglycemic clamp. **(B)** Blood glucose levels. (# p<0.0001, LOW versus HIGH group for the indicated time points). **(C)** Glucose infusion rates. **(D)** Serum ferret insulin levels during clamp studies. (* p<0.05 both versus 120 minutes and LOW versus HIGH; # p<0.01 versus baseline.) The HIGH group insulin levels were measured by Calbiotech insulin ELISA assay that also reacts with insulin lispro. The LOW group insulin levels were measured by immunobead fluorescence multiplex assay that does not recognize insulin lispro. This immunobead assay was used to achieve greater sensitivities for lower insulin levels. **(E)** Fluorescence intensities resulting from regular and lispro insulin using an immunobead fluorescence multiplex assay. See Supplemental Figure S2 for additional details.

Glycemic clamps were performed to assess insulin secretion during controlled hyperglycemia and hypoglycemia. Ferrets were conscious and minimally-restrained while undergoing 180 minute clamp. The data are shown in three groupings. All ferrets underwent hyperglycemic clamp (HG) for the first 120 minutes. A portion of these ferrets were maintained at hyperglycemia for the 120–180 min period (“HIGH”), whereas the remainder underwent hypoglycemic clamp during the 120–180 min period (“LOW”) (Figure 3A). Weights were similar between the HIGH and LOW groups at 232±8 (n = 6) and 243±8 (n = 7) respectively. Ferrets reached the goal level of hyperglycemia by 10 minutes, and hyperglycemia was maintained thereafter (Figure 3B) in both the HG and HIGH groupings. Approximately 20 mg/kg/min glucose infusion was required to maintain this degree of hyperglycemia (Figure 3C). Under these conditions, a first phase insulin secretory response was evident, with a significant rise in insulin occurring by 2.5 minutes and peaking at 10–20 minutes after initiation of the glucose infusion (Figure 3D). Insulin levels diminished modestly thereafter to a plateau maintained for 2 hours but further diminishing thereafter.

Glucose levels rapidly diminished after 120 min in the LOW but not HIGH ferret group (Figure 3A). Hypoglycemia was achieved by 30 minutes in the LOW group and glucose infusion was required to avoid further glucose reductions (Figure 3B). To assay endogenous ferret insulin but not exogenous lispro insulin in these ferrets, we identified two assays that detected ferret insulin but not insulin lispro. This immunodistinction is possible because the B-chain of ferret insulin differs from insulin lispro at 3 amino acid residues, but differs from human insulin only at 1 residue and is identical to porcine and canine insulin (Table 2). An immunobead fluorescence multiplex assay detected regular insulin but not insulin lispro (Figure 3E). This assay showed that ferret insulin diminished rapidly in the LOW group during hypoglycemia to sub-baseline levels (Figure 3D). As a confirmatory approach, we employed a porcine insulin ELISA kit (Mercodia Porcine insulin) known to not react with insulin lispro. As expected based on the identical amino acid sequences of pig and ferret insulin, we found that the ELISA recognized ferret but not lispro insulin (Supplemental Figure S2C). Assay with this ELISA showed results nearly identical to the immunobead assay, with insulin levels rapidly diminished in the LOW ferrets during hypoglycemia (Figure 3D). The entire insulin time course for the HIGH and LOW groups separately, including determination of the LOW group insulin levels using both the multiplex and the porcine insulin assays, is detailed separately in Supplemental Figure S2 A,B,D.

**Table 2:** Comparative insulin B-chain amino acid sequences by species.

Species	Sequence a
Ferret	FVNQHLCGSHLVEALYLVCGERGFFYTPKA
Human	FVNQHLCGSHLVEALYLVCGERGFFYTPK**T**
Lispro	FVNQHLCGSHLVEALYLVCGERGFFYT**KPT**
Mouse(1)	FV**K**QHLCG**P**HLVEALYLVCGERGFFYTPK**S**
Mouse(2)	FV**K**QHLCGSHLVEALYLVCGERGFFYTP**MS**
Dog	FVNQHLCGSHLVEALYLVCGERGFFYTPKA
Pig	FVNQHLCGSHLVEALYLVCGERGFFYTPKA
Cat	FVNQHLCGSHLVEALYLVCGERGFFYTPKA

a Differences in sequence from that of ferret are bolded. The ferret insulin Ensembl gene identification is ENSMPUG00000005638.

### Islet and Entero-insular hormonal responses to hyper- and hypoglycemia

**Figure 4:**
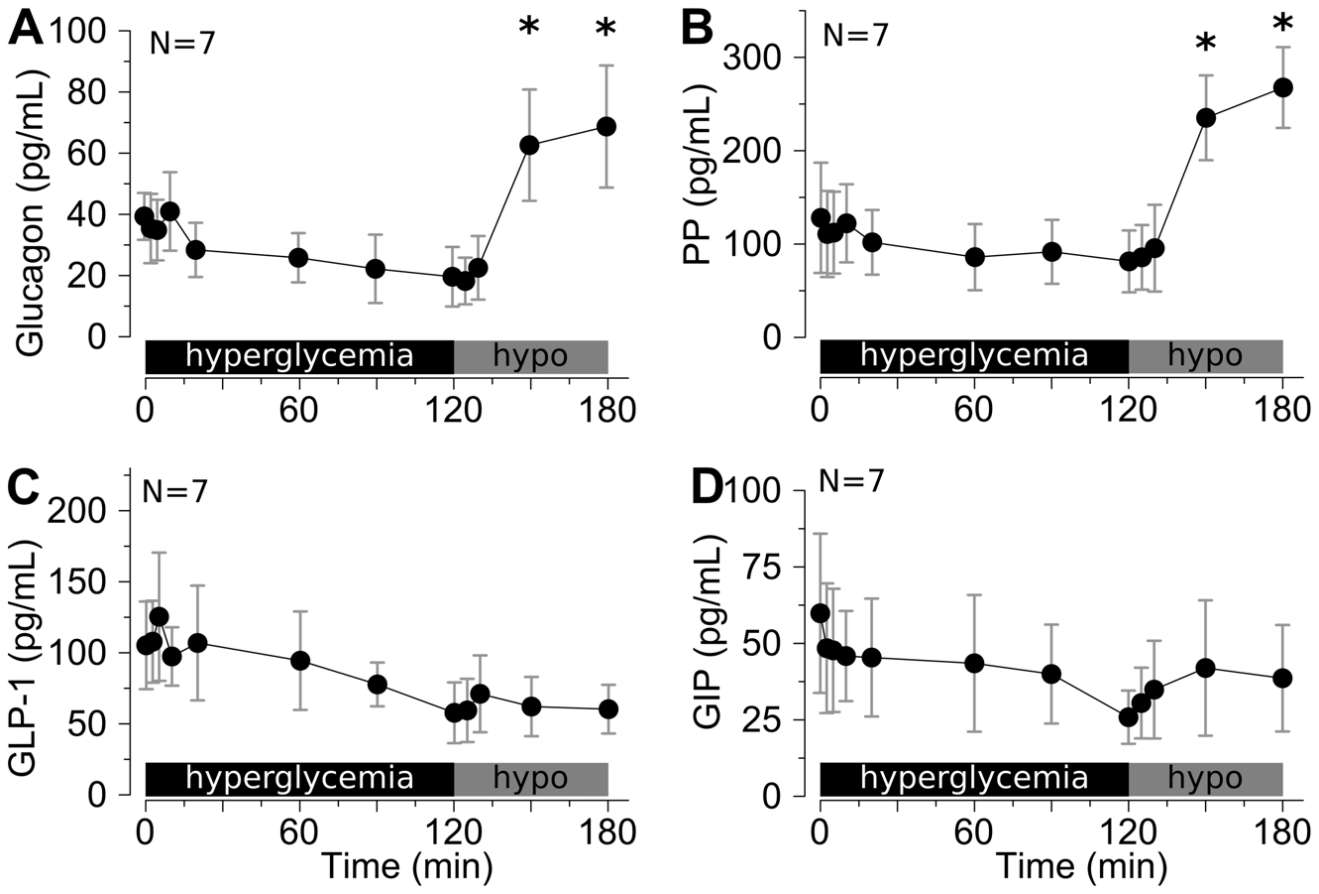
Islet and incretin hormonal responses to hyper- and hypoglycemia in ferrets. Serum samples were from the hyperglycemic→hypoglycemic clamps shown in Figure 3. **(A)** Glucagon, **(B)** pancreatic polypeptide (PP), **(C)** GLP-1, and **(D)** GIP were measured. * p<0.05 versus 120 min time-point (N = 7). There were no significant changes compared to baseline during hyperglycemia.

Glucagon levels showed no statistical change despite a modest trend towards decreases in value with hyperglycemia. Glucagon levels were significantly increased by hypoglycemia (Figure 4A). Pancreatic polypeptide levels showed a nearly identical response pattern as glucagon (Figure 4B). By contrast, GLP-1 and GIP levels exhibited no significant changes during changing glycemia, though both drifted towards lower levels with time (Figures 4 C,D).

## Discussion

This is the first report, to our knowledge, of glycemic clamps in ferrets. Glycemic clamps are an ideal means to assess insulin sensitivity and insulin secretion. Ferrets serve as a useful model of various human diseases including CFRD [22]. Ferrets thus represent a unique opportunity to understand the pathophysiological underpinnings of CFRD. Ferrets might be expected to exhibit some metabolic differences from humans, due to the fact ferrets are carnivores and humans are omnivores. For example, ferrets are thought to be obligate carnivores and thrive on a high-protein, high-fat, low-carbohydrate diet [31]. Furthermore, transit time through the ferret gut is rapid. Nonetheless, we find that like humans and other mammals, ferrets exhibit whole-body responsiveness to insulin and exhibit a first phase peak of insulin secretion in response to sustained hyperglycemia.

In our ferret study, infusion of 4 mU/kg/min insulin achieved modest steady state insulin levels of ∼25–150 µU/mL and required glucose infusion rates of 10–30 mg/kg/min to maintain euglycemia. Studies in conscious rats yield similar parameters, whereby infusion of 1.2–24 mU/kg/min insulin yield insulin levels of 40–1300 µU/mL and require a glucose infusion rate of 8 to 35 mg/kg/min to maintain euglycemia [31]. In humans, infusion of 0.2–2.0 mU/kg/min insulin requires glucose infusion rates of 1–9 mg/kg/min to maintain euglycemia [33]. Not surprisingly, infusion of 20 mU/kg/min compared to 4 mU/kg/min insulin into ferrets induced higher insulin levels and produced a requirement for a faster glucose infusion rate at ∼40 mg/kg/min to maintain euglycemia. The maximal dose response in humans occurs by insulin concentrations of ∼10,000 µU/mL [34]. Thus, it is likely that the insulin infusion rates used in our ferret euglycemic hyperinsulinemic clamps represents a moderate dose, and that lesser and greater degrees of glucose infusion would be required by lower or higher insulin doses. In cats, infusion of 10 mU/kg/min required glucose infusion rate of 8 mU/kg/min [35], leading to the suggestion cats may be relatively insulin resistant compared to non-carnivorous species [36]. Our data indicate that the insulin resistance observed in cats is not shared with the ferrets in this study, though both are carnivorous domestic species. However, definitive conclusions are prevented by differences in the species of origin of infused insulin and imperfect correlation between differing insulin assays.

We found that glucose tracing approaches, just as in humans and other animal models, can be used with euglycemic hyperinsulinemic clamp in the ferret, allowing deconvolution of glucose disposal and glucose appearance rates. In these studies, glucose appearance was sensitive to insulin such that even lower dose infusion (4 mU/kg/min) was completely suppressive. By contrast, glucose disposal was further stimulated by greater degrees of insulin infusion. This dichotomy between the concentration dependence of insulin responsiveness of glucose disposal versus appearance is similar to that of other species [32].

In at least some ferret populations, those above age 4–5 years have an approximately 25% incidence of insulinomas, most of which appear to be malignant [19], [21]. Importantly, the ferrets used in this study were far younger, at 5–6 weeks. The etiology for the high prevalence of insulinomas in ferrets remains unknown. However, the finding does reinforce that ferret beta-cells may be highly sensitive to injuries that lead to transformation. Whether this has relevance to the ability of the ferret to model CFRD remains unclear. A portion of the anesthetized and high-dose clamps exhibited less than ideal glycemic stability, marked by a drop in glucose early in the clamp. With increasing experience, these early dips likely can be prevented by greater degrees of glucose infusion early during the clamp.

In our ferret study, acute elevation of glucose from 125 to ∼250 mg/dL led to an acute insulin secretory response to 20 µU/mL with a peak ∼10 minutes. The observed timing of insulin response to hyperglycemia clamp is similar to the pattern observed in other species. In humans, a rise in glucose from ∼90 to ∼215 produced an increment in insulin from 10 to 60 µU/mL [24]. In rats, a rise in glucose from ∼125 to 250 mg/dL produces an insulin increment rising from 6 to 80 µU/mL peaking by 10 minutes [37]. In cats, a rise in glucose from ∼110 to ∼160 mg/dL produces an insulin increment rising from 11 to 33 µU/mL peaking at 10 minutes [36]. Thus, although the timing of first phase insulin secretion in these ferrets is similar to that observed in other species, the amplitude of the response is more similar to that observed in cats [36], also a carnivore, being less than that observed in humans and rats.

Our cortisol data suggest that in general the ferrets remained relatively unstressed during awake clamps. A portion of ferrets exhibited higher cortisol levels, but none exhibited persistent abnormal elevations and furthermore cortisol levels did not influence insulin sensitivity as measured by glucose infusion rate. By contrast, insulin sensitivity was dramatically reduced in isoflurane-anesthetized ferrets, with the required glucose infusion rate being 35% of that of conscious ferrets. In fact, higher insulin levels were achieved during the clamp in ANESTH compared to AWAKE ferrets, indicating an even greater degree of insulin resistance had the insulin levels been identical between groups. Isoflurane-induced insulin resistance may be related to increased epinephrine levels, diminished cerebral glucose utilization, or increased hepatic glucose production [38], [39]. The marked elevation of insulin levels during the hyperinsulinemic clamp in isoflurane treated ferrets suggests that the observed insulin resistance involves the liver, given that hepatic insulin resistance typically results in impaired hepatic insulin clearance and ensuant hyperinsulinemia.

Ferrets exhibited rapid decreases in insulin levels in response to hypoglycemia that followed hyperglycemia, similar to but perhaps more rapid and profound than that observed in similar studies in humans [40]. This finding and approach will allow tests for possible dysfunctional responses of the ferret insulin-axis to hypoglycemia in CF. Our studies showed that hypoglycemia following hyperglycemia in ferrets produces increases in glucagon and pancreatic polypeptide, similar to that observed in human studies [41]. By contrast, GLP-1 and GIP are not significantly affected by circulating hyperglycemia in the ferret [42]. In humans, neither GLP-1 and GIP undergo much change in response to hyperinsulinemia-induced hypoglycemia [29], [43]. Similar to our findings in ferrets, in humans very modest decreases in glucagon [44] and no change in pancreatic polypeptide [45] occur during hyperglycemic clamp. We find no published data on hypoglycemic counterregulation or neuroglycopenia in the ferret, and our studies were not designed to assess these responses or thresholds.

In summary, we described the use of glycemic clamps to assess insulin sensitivity and entero-insular hormonal responses in response to hyper- and hypoglycemia in ferrets. Ferrets exhibit sensitivity to insulin that is similar to rodents and humans and insulin secretion similar to that observed in cats. Ultimately these techniques will be useful in unraveling the pathophysiology of CFRD in ferrets.

## Supporting Information

Figure S1 **Correlations between serum cortisol and glycemic parameters.** **(A)** Relation between baseline and end-of-clamp serum cortisol levels and the minimum and maximum observed glucose levels during clamp. Bonferroni corrected and nominal (i.e. uncorrected, in parentheses) p-values for Pearson's correlation shown. **(B)** Relation between baseline and end-of-clamp serum cortisol levels and the end-of-clamp glucose infusion rate (GIR). Nominal p-values for Pearson's correlation shown.
(PNG)

Figure S2 **Additional details regarding insulin measurement during non-euglycemic clamp studies.** **(A)** Serum ferret insulin levels during non-euglycemic clamp studies, replotting of Figure 3D, separating the HIGH and LOW groups at all time points. **(B)** LOW group data only, using the Milliplex insulin assay. **(C)** Absorbance intensities resulting from ferret and lispro insulin using a porcine ELISA insulin assay. **(D)** LOW group data only, using the porcine ELISA insulin assay (Mercodia). (A,B,D) # p<0.05 versus respective zero time point; * p<0.05 versus respective 120 in time point. Note that the LOW group insulin levels are significantly below baseline at the end of the clamp, while the HIGH group insulin levels remain significantly higher than baseline.
(PNG)
