# Supplementary figures and images for: Quantifying Insulin Sensitivity and Entero-Insular Responsiveness to Hyper- and Hypoglycemia in Ferrets
Source: PLoS One. 2014 Mar 3;9(3):e90519. doi: 10.1371/journal.pone.0090519 (PMC3940889; doi:10.1371/journal.pone.0090519)

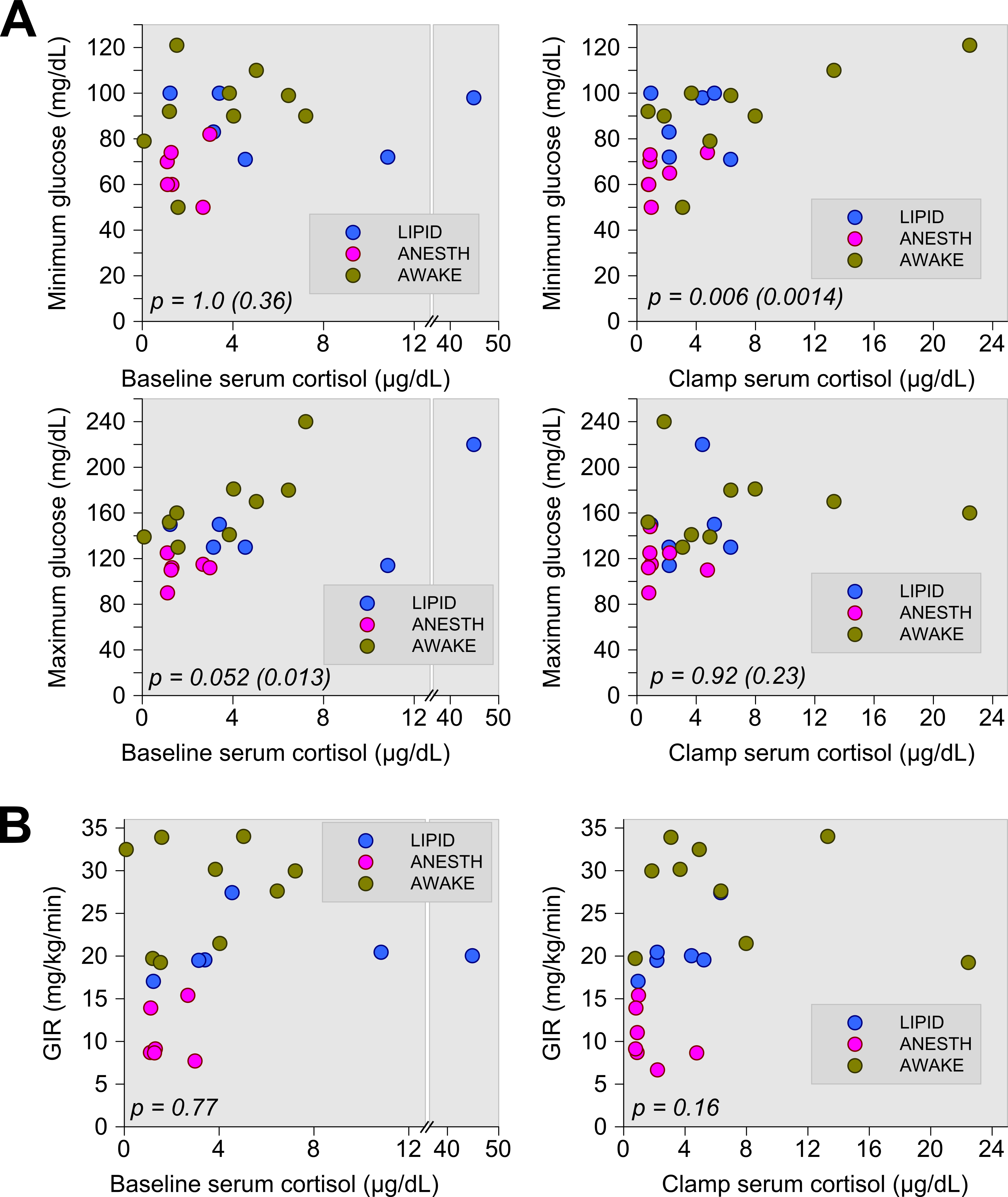

Supplement: Figure S1 — Correlations between serum cortisol and glycemic parameters. (A) Relation between baseline and end-of-clamp serum cortisol levels and the minimum and maximum observed glucose levels during clamp. Bonferroni corrected and nominal (i.e. uncorrected, in parentheses) p-values for Pearson's correlation shown. (B) Relation between baseline and end-of-clamp serum cortisol levels and the end-of-clamp glucose infusion rate (GIR). Nominal p-values for Pearson's correlation shown. (PNG) [file pone.0090519.s001.png]

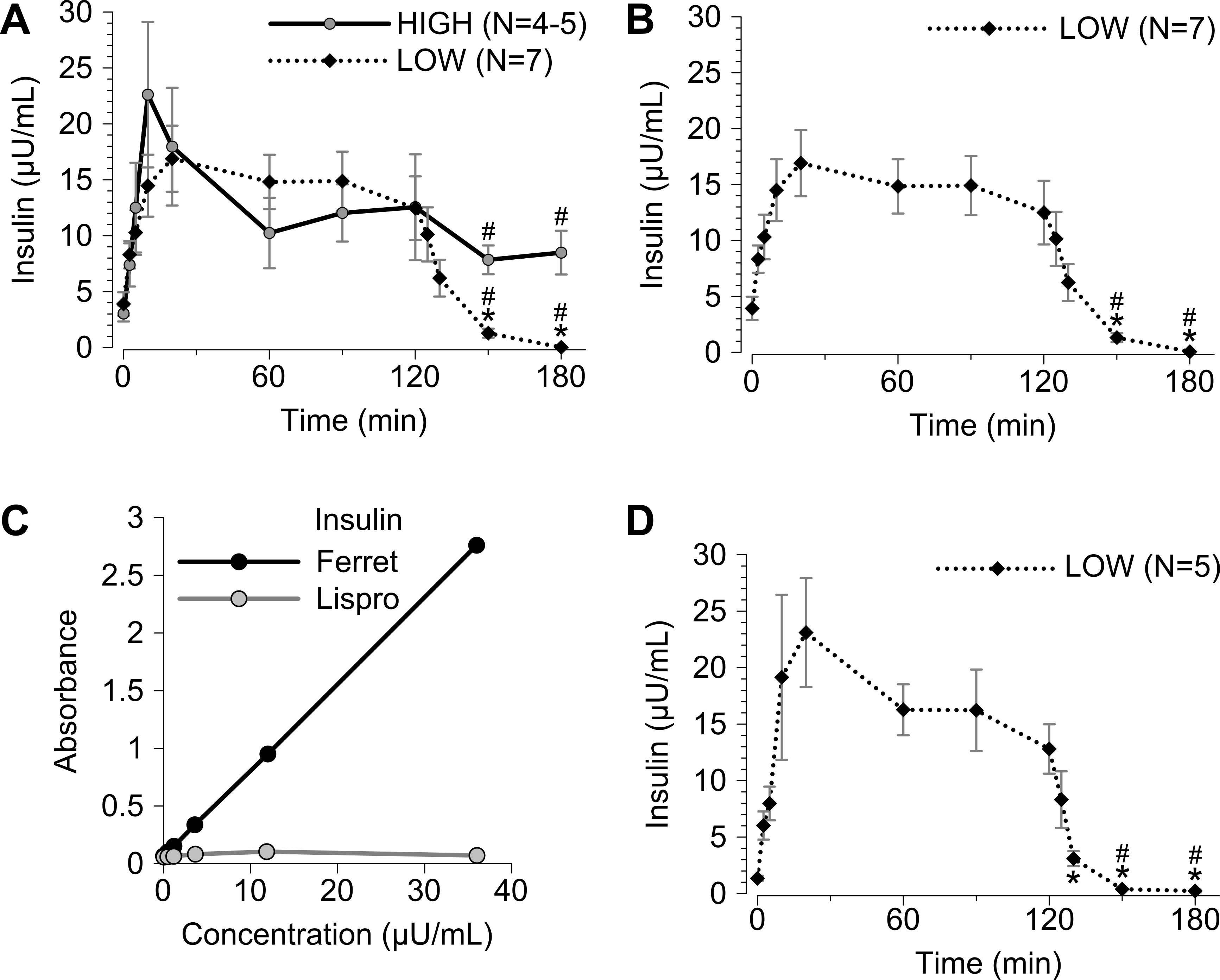

Supplement: Figure S2 — Additional details regarding insulin measurement during non-euglycemic clamp studies. (A) Serum ferret insulin levels during non-euglycemic clamp studies, replotting of Figure 3D, separating the HIGH and LOW groups at all time points. (B) LOW group data only, using the Milliplex insulin assay. (C) Absorbance intensities resulting from ferret and lispro insulin using a porcine ELISA insulin assay. (D) LOW group data only, using the porcine ELISA insulin assay (Mercodia). (A,B,D) # p<0.05 versus respective zero time point; * p<0.05 versus respective 120 in time point. Note that the LOW group insulin levels are significantly below baseline at the end of the clamp, while the HIGH group insulin levels remain significantly higher than baseline. (PNG) [file pone.0090519.s002.png]
